# Effects on abstinence of nicotine patch treatment before quitting smoking: parallel, two arm, pragmatic randomised trial

**DOI:** 10.1136/bmj.k2164

**Published:** 2018-06-13

**Authors:** The Preloading Investigators

**Affiliations:** Nuffield Department of Primary Care Health Sciences, University of Oxford, Oxford OX2 6GG, UK

## Abstract

**Objective:**

To examine the effectiveness of a nicotine patch worn for four weeks before a quit attempt.

**Design:**

Randomised controlled open label trial.

**Setting:**

Primary care and smoking cessation clinics in England, 2012-15.

**Participants:**

1792 adults who were daily smokers with tobacco dependence. 899 were allocated to the preloading arm and 893 to the control arm.

**Interventions:**

Participants were randomised 1:1, using concealed randomly permuted blocks stratified by centre, to either standard smoking cessation pharmacotherapy and behavioural support or the same treatment supplemented by four weeks of 21 mg nicotine patch use before quitting: “preloading.”

**Main outcome measures:**

The primary outcome was biochemically confirmed prolonged abstinence at six months. Secondary outcomes were prolonged abstinence at four weeks and 12 months.

**Results:**

Biochemically validated abstinence at six months was achieved by 157/899 (17.5%) participants in the preloading arm and 129/893 (14.4%) in the control arm: difference 3.0% (95% confidence interval −0.4% to 6.4%), odds ratio 1.25 (95% confidence interval 0.97 to 1.62), P=0.08 in the primary analysis. There was an imbalance between arms in the frequency of varenicline use as post-cessation treatment, and planned adjustment for this gave an odds ratio for the effect of preloading of 1.34 (95% confidence interval 1.03 to 1.73), P=0.03: difference 3.8% (0.4% to 7.2%). At four weeks, the difference in prolonged abstinence unadjusted for varenicline use was odds ratio 1.21 (1.00 to 1.48), difference 4.3% (0.0% to 8.7%), P=0.05, and adjusted for varenicline use was 1.32 (1.08 to 1.62) P=0.007. At 12 months the odds ratio was 1.28 (0.97 to 1.69), difference 2.7% (−0.4% to 5.8%), P=0.09 unadjusted for varenicline use and after adjustment was 1.36 (1.02 to 1.80) P=0.04. 5.9% of participants discontinued preloading owing to intolerance. Gastrointestinal symptoms—chiefly nausea—occurred in 4.0% (2.2% to 5.9%) more people in the preloading arm than control arm. Eight serious adverse events occurred in the preloading arm and eight in the control arm (odds ratio 0.99, 0.36 to 2.75).

**Conclusions:**

Evidence was insufficient to confidently show that nicotine preloading increases subsequent smoking abstinence. The beneficial effect seems to have been masked by a concurrent reduction in the use of varenicline in people using nicotine preloading, and future studies should explore ways to mitigate this unintended effect.

**Trial registration:**

Current Controlled Trials ISRCTN33031001.

## Introduction

Although there have been several new drugs for tobacco cessation since the 1970s, the paradigm of treatment has remained largely the same, with no major advances in success rates. Treatment comprises behavioural support to motivate and strengthen a person’s resolve to remain abstinent and drugs to reduce the strength of urges to smoke after quit day.

Most adult smokers want to stop smoking but continue because they have developed a drive to smoke. Repeated pairing of smoking behaviour with stimulation of cholinergic receptors in the midbrain that project to the nucleus accumbens is an important factor contributing to this drive.^1^ The urge to smoke can remain largely unconscious, but when a smoker decides to quit a craving is experienced, typically in response to moods or situations when smoking would normally have occurred. These cravings undermine the success of most quit attempts.^2^ Effective smoking cessation drugs exert their effect by reducing the intensity of cravings, and the most effective drug currently available, varenicline, does so to the greatest extent.^3^ In people who resist the craving, repeated exposure to cues to smoke without actually smoking and the concomitant delivery of nicotine from a cigarette will mean the drive to smoke is unlearnt and craving intensity decreases.

If smoking occurs without the reinforcement provided by nicotinic stimulation, the drive to smoke should diminish and, when a smoker attempts to quit, they should experience less intense craving and be more likely to succeed. One way to block the effect of nicotine from cigarettes is to use a nicotine patch. Doing so desensitises the cholinergic brain receptors, meaning they are refractory to further stimulation from cigarette supplied nicotine.^4^ This should reduce the intensity of the urge to smoke while smoking and, crucially, lowered dependence will reduce craving intensity if someone makes a quit attempt, facilitating abstinence.

Using a nicotine replacement treatment (or other smoking cessation drugs) before a quit attempt while smoking normally is called preloading. A systematic review showed some, but not convincing, evidence that nicotine preloading may be effective, with much unexplained heterogeneity between studies.^5^ Some studies suggested that nicotine preloading doubled the likelihood of achieving abstinence, which, if true, suggests that using nicotine replacement therapy before a quit attempt is more effective than when used in the conventional way to support smoking abstinence.^6^ Other studies suggested preloading had no effect. A partial explanation for the heterogeneity may relate to the form of nicotine replacement used. Smoking while using patches produces higher blood nicotine concentrations than just smoking, but smoking while using short acting nicotine replacement therapy, such as gum, does not.^7^ There was some but not strong evidence in the review that preloading using a nicotine patch was more effective than preloading with oral nicotine replacement therapy. Since then, three more studies using varenicline and bupropion preloading have been conducted, with some evidence of a benefit on short term cessation.^8^ ^9^ ^10^ These studies are relevant because they examine the same hypothetical active ingredient of the preloading interventions as studies of nicotine replacement therapy—that is, the effects of reduced satisfaction from smoking. Together, these studies have provided modest support that preloading works through reducing the intensity of urges to smoke.^78-12^

Given the promise and uncertainty around this novel approach to treating tobacco dependence, we conducted a large trial in a routine health service context to examine the impact of nicotine replacement therapy preloading on long term abstinence and mechanisms of its action.

## Methods

This was an open label multicentre pragmatic superiority trial, with participants randomised 1:1 to receive or not receive a nicotine patch to use for four weeks before quit day. Thereafter the participants used standard pharmacotherapy and behavioural support to support cessation. The primary outcome was prolonged biochemically validated abstinence measured six months after quitting. The protocol is published and was implemented with one change^11^; we asked participants who had moved house and were unable to attend in person to return a supplied saliva swab to measure cotinine or anabasine concentrations to confirm abstinence. The planned cost effectiveness analysis will be presented separately.

### Participants and settings

In three recruitment centres, based in Nottingham, Birmingham, and Bristol, general practitioners spoke or wrote to, emailed, or texted patients listed as smokers on the electronic health record and invited them to join the trial as a means to stop smoking. The fourth recruitment centre, in London, was an existing National Health Service smoking cessation clinic and invited patients seeking treatment to participate in the trial.

Potential participants telephoned the research team to learn more about the trial and were screened for eligibility. If they seemed to be eligible and wanted to participate, we booked them in for an appointment at their general practice or smoking cessation clinic to meet the researcher, and we sent them an information sheet. At this initial appointment, we again described the trial and obtained consent.

Potential participants were eligible for the study if they regularly smoked cigarettes, cigars, or roll-up tobacco cigarettes, with or without marijuana; were aged 18 years or more; would be suitable for preloading according to researcher’s judgment; were seeking support to stop smoking from the NHS stop smoking service; were willing to set a quit day in four weeks; and were able to understand and willing to adhere to study procedures. We excluded those who were pregnant or breast feeding; had skin disorders that precluded use of the patch; had acute coronary syndrome or stroke in the past three weeks; and had an active phaeocromocytoma or uncontrolled hyperthyroidism that would increase the risk of arrhythmias from the nicotine patch.

To determine suitability for preloading we used several criteria with no cut-offs to assess whether potential participants were addicted to smoking: failure of previous quit attempts despite use of appropriate pharmacotherapy; time to first cigarette in the morning, with earlier use reflecting higher addiction; number of cigarettes smoked daily, with a greater number reflecting higher addiction; and exhaled carbon monoxide concentration, with higher values reflecting higher addiction.

### Interventions

#### Preloading arm

We asked participants in the preloading arm to use a 21 mg/24 h nicotine patch daily for approximately four weeks before quit day. If participants had had problems with overnight use in previous quit attempts, we advised wearing the patch in waking hours only.

We asked participants to smoke as normal and not reduce consumption. Reduced consumption would probably lower blood nicotine concentration, which could make cigarettes more rewarding and thus undermine the supposed benefits of preloading.^13^

Participants in both arms were referred to the NHS stop smoking service for continuing support with cessation. We asked participants and the NHS advisors by letter and in person to set a quit date between three and five weeks after commencing preloading. We allowed that preloading could continue for up to eight weeks in exceptional circumstances, and also that preloading could restart once—for example, if preloading was interrupted because the participant was admitted to hospital. In such cases, participants aimed to complete three to five weeks of preloading from the date of recommencement.

At the first visit the researcher explained the rationale of preloading and prompted action planning to maximise adherence to the patches. The researcher addressed participants’ concerns about smoking while using patches and advised on how to manage side effects. The researcher talked through the participant’s daily routine and noted opportunities to use environmental cues to minimise the chance of forgetting to apply the patch daily. To reinforce this, we provided a booklet with the same information. Participants commenced preloading at this visit and we reviewed them one week later to deal with concerns and reinforce adherence.

We offered participants lower strength patches at commencement if they reported previous adverse reactions to the 21 mg patch, or during the treatment course if they experienced symptoms of nicotine overdose such as nausea, salivation, and pounding heart. Preloading was stopped if requested by participants, it was not possible to alleviate adverse events by reducing the dose, or an intervening health state or contraindication to preloading emerged.

#### Control arm

We aimed to balance participants’ expectations of success and to assess adverse events in an unbiased way. A placebo would have achieved this but owing to funding restrictions we developed a behavioural intervention. We asked participants to consider their smoking pattern, to consider the triggers for use of particular cigarettes, and to plan ways to reduce these cues. This is standard in smoking cessation support anyway, so participants in the preloading arm may well have done this later when they enrolled in their NHS stop smoking service. Participants in the control arm received a booklet outlining this process, which was similar in length and appearance to the booklet supplied to participants in the preloading arm. As in the preloading arm, participants in the control arm attended and received this support at baseline and one week later and were also referred to the NHS stop smoking service to commence a quit attempt between three and five weeks after enrolment.

### Standard smoking cessation treatment

At the first and second visits in both arms, we referred participants to their local stop smoking service, and we wrote a letter to the advisors to ask them to encourage participants to continue preloading. We asked advisors to ignore the presence or absence of nicotine patch treatment when choosing pharmacotherapy to support the quit attempt. We were concerned that NICE guidance recommends against the concurrent use of nicotine replacement therapy and varenicline (which is started a week before quit day),^14^ which advisors often assumed was because of issues around safety. We tried to correct misconceptions in the referral letter, by telephone, and face-to-face discussions with clinicians.

The advisors from NHS stop smoking service provided weekly behavioural support starting one or two weeks before quit day and continuing until at least four weeks after quit day, and they encouraged participants to maintain total abstinence from quit day onwards. This support involves planning for the quit day; explanation of how occasional lapses can undermine quitting; encouragement to adopt a “not a puff” rule; supervision and facilitation of drug use; advice on and how to deal with temptations to smoke; and monitoring of abstinence through carbon monoxide testing. The support is termed withdrawal oriented therapy.^15^

### Measures and outcomes

At baseline we recorded participants’ smoking history and basic demographic information. This included two markers of cigarette dependence: the Fagerstrom test for cigarette dependence, which measures consumption of cigarettes and difficulty in refraining from smoking, and the concentration of exhaled carbon monoxide.^16^

We followed-up participants on five occasions. Participants returned one week after the baseline appointment for the −3 week appointment (about three weeks before quit day). Occurrence of adverse events and adherence to preloading was assessed. We telephoned participants one week after quit day (+1 week), about six weeks after baseline, to collect data on adverse events, adherence to preloading, and use of and adherence to other smoking cessation pharmacotherapy. We obtained data on smoking cessation from the NHS stop smoking service or the participant at +4 weeks. At six and 12 months we telephoned participants to obtain data on smoking status and health service use. To confirm abstinence biochemically we invited participants who were abstinent to attend to measure the concentration of carbon monoxide in end expiratory air using a handheld device.

#### Primary outcome and secondary outcomes

The primary outcome was prolonged abstinence at six months, defined by the Russell standard criteria.^17^ This allows a grace period of two weeks after quit day, when lapses do not count against abstinence. Thereafter we counted a person as abstinent if they smoked fewer than five cigarettes to the assessment at six months and were confirmed as abstinent if an exhaled carbon monoxide concentration was <10 ppm.

The secondary outcomes were Russell standard abstinence at four weeks and 12 months, and biochemically confirmed seven day point prevalence abstinence at four weeks and six and 12 months.

### Intensity of urges to smoke

As the principal hypothesised mechanism of action is that preloading undermines the intensity of urges to smoke, we examined the effect of preloading on this, measured at −3 weeks (while using preloading or as control) and at +1 week after quit day using the mood and physical symptom scale-craving subscale (scored 0-5, with higher scores representing more severe symptoms). In the latter assessment, in accord with consensus,^18^ the analysis was confined to those who were abstinent or still trying to achieve abstinence.

### Adverse events

Adverse events were defined as newly occurring health conditions or exacerbations of existing conditions. We recorded adverse events that were either serious or of moderate or severe intensity between baseline and one week after quit day, covering the period of preloading and allowing one additional week for adverse events to emerge. Moderate or severe adverse events were defined as those that interfered somewhat or totally with normal functioning. Serious adverse events were defined as resulting in admission to hospital, death or life threatening events, permanent disability, or congenital abnormality. This excluded planned events, such as scheduled surgery. An independent committee assessed serious adverse events and adjudicated whether the event was unrelated, unlikely, possibly, probably, or definitely related to the use of nicotine patches. The committee members were blind to treatment allocation and hence also the temporal relation of drug use to the event.

We elicited adverse events from participants in both arms at contacts −3 weeks and at +1 week by asking about new or worsening health problems, followed by further inquiry as appropriate. These were coded using MedDRA v19.1. As it can be hard for trial staff and participants to understand the necessity to report adverse events for people in the control arm receiving no treatment, we also gave participants in both arms a questionnaire to complete about symptoms. This assessed symptoms of nicotine overdose in the previous 24 hours (such as nausea, excessive salivation) one week after baseline. Participants rated how troubled they had been by the symptoms in the past 24 hours on a scale from “not at all” to “very” or “extremely.”

### Sample size, randomisation, and blinding

Based on data from similar trials we estimated that 15% of participants in the control arm would achieve abstinence at six months.^19^ ^20^ ^21^ We thought that a relative risk of 1.4 was both plausible and valuable for patients, implying a 6% absolute difference.^5^ This gave us a sample size of 893 in each study arm or 1786 in total, to achieve 90% power using χ^2^ test with Yates’ correction.

An independent statistician used Stata to generate a randomisation list stratified by treatment centre and using randomly permuted blocks of varying size in a 1:1 ratio. This was incorporated into an online database, and the sequence remained concealed from research staff until they had entered data to allow randomisation.

This was an open label trial so participants, research staff, and NHS stop smoking service staff knew the arm to which participants were assigned. Blinded follow-up was impractical because staff had been involved in recruitment, but abstinence was biochemically confirmed.

### Statistical analysis

We followed the Russell standard approach to perform an intention to treat analysis for the abstinence outcome.^17^ Everyone randomised was included in the denominator, whenever and however smoking abstinence was assessed, and they were presumed to be smoking if this information was unknown. In the primary analysis, we calculated adjusted odds ratios using multivariable logistic regression in Stata 14.2 adjusted for the stratification variable (centre). We also calculated the percentage of participants achieving abstinence, the risk difference and risk ratios and 95% confidence intervals using the post-estimation adjrr procedure in Stata v14.2.

In planned sensitivity analysis, we adjusted for two predictors of abstinence to improve precision: longest previous abstinence and degree of addiction measured by strength of urges to smoke at baseline.^22^ ^23^ ^24^ Secondly, varenicline is more effective than other pharmacotherapy and is commonly used to assist with cessation.^25^ As we anticipated that using nicotine preloading in an open label trial could deter use of varenicline, we adjusted for use of varenicline after quit day to overcome confounding.

We planned subgroup analyses by including multiplicative interaction terms in the equations. The presumed mechanism of action of preloading suggests greater benefit from preloading in people more dependent on cigarettes. We used two markers of dependence: baseline Fagerstrom test for cigarette dependence score and exhaled carbon monoxide concentration added as continuous terms. We also examined whether the effect of preloading was less pronounced in participants using varenicline. Normal use of varenicline involves a week of use before quit day and there is evidence that this may have similar effects to preloading, which could undermine the effect of nicotine preloading.^8^

Using a linear regression model with adjustment for baseline mood and physical symptom scale-craving subscale score and stratification variables, we analysed the effect of preloading on urge to smoke (mood and physical symptom scale-craving subscale).

The denominator for analysis of adverse events was all those who provided data on such events. The analysis used analogous statistical models to those applied for the primary and secondary outcomes.

### Patient involvement

The idea for the study and the proposed methods were discussed by us in a meeting with the UK Centre for Tobacco and Alcohol Studies smokers panel. This panel is a standing group of people who smoke or have recently stopped smoking, and they agreed the study was valuable and gave views on the design that shaped the protocol. After early initial slow recruitment, we returned to eight members of the panel to discuss the letter from general practitioners to potential participants and participant information leaflet and made some minor amendments as a result. One member of the panel also served on the independent trial steering committee.

## Results

**Fig 1:**
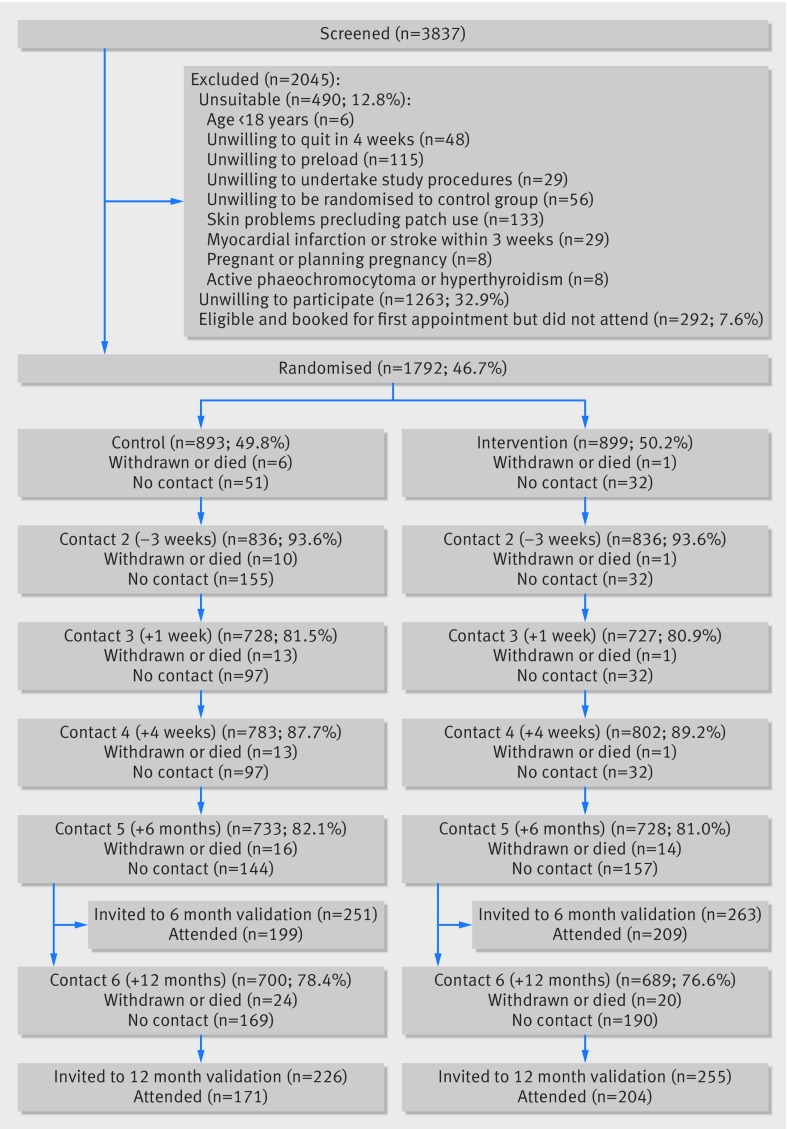
CONSORT flow diagram. 893 control participants and 899 intervention participants were analysed for all primary and secondary outcomes, and those whose true status was unknown were counted as smokers

Between 13 August 2012 and 10 March 2015, 3837 people were telephoned about enrolment. In total, 490 (12.8%) were ineligible, most commonly owing to skin problems or unwillingness to use preloading patches. Of 1805 (47.0%) patients seen at the initial appointment, 1792 (99.3%) were eligible and enrolled (fig 1).

One week after baseline we followed-up 1702 (95.0%) participants, and five weeks after baseline, one week after quit day, we obtained data from 1456 (81.3%) participants. These assessments provided data on adverse events.

We obtained abstinence data on 1585 (88.5%) participants at four weeks, 1461 (81.5%) at six months, and 1389 (77.5%) at 12 months. A similar proportion was successfully followed in each arm. Although 331 (18.5%) participants were not available for the primary outcome assessment at six months, we knew that 97 were smoking at four weeks and that 54 never made a quit attempt; thus neither group could be classified as abstinent at six months. We were therefore certain of the primary outcome in 1612 (90.0%) participants.

### Baseline characteristics

Most participants were middle aged, half were men, and a quarter were from minority ethnic groups. The participants had lower levels of educational attainment than the UK average,^26^ and half were employed. Participants smoked a mean of 18.9 (SD 9.3) cigarettes/day at baseline, and had a mean dependence score indicating moderate addiction and a mean exhaled carbon monoxide concentration of 23.7 (SD 12.5) ppm. A third had used behavioural support or pharmacotherapy to try to quit in the past six months. Baseline characteristics were well balanced between trial arms (table 1). The main predictors of abstinence are markers of cigarette dependence, such as the Fagerstrom test for cigarette dependence and exhaled carbon monoxide concentration, and these were almost the same between arms. Likewise, demographic variables were balanced, with mean age differing between arms by around a third of a year, the same proportion of males, and the same proportion of people from ethnic groups who were not white British. Education and employment were also balanced between trial arm, with less than 2% difference between the proportions in any category of these variables. The biggest difference appeared in the proportion of people who had used cessation aids in the past six months: 31.0% in the preloading arm and 34.0% in the control arm.

**Table 1:** Participant characteristics by trial arm. Values are numbers (percentages) unless stated otherwise

Characteristics	Control arm (n=893)	Preloading arm (n=899)	Total (n=1792)
Mean (SD) age (years)	48.8 (13.4)	49.1 (13.3)	48.9 (13.4)
Men	469 (52.6)	473 (52.6)	942 (52.6)
Women	422 (47.3)	426 (47.4)	848 (47.4)
Ethnicity:			
White British	675 (75.6)	680 (75.6)	1355 (75.6)
White Irish	36 (4.0)	25 (2.8)	61 (3.4)
White other	57 (6.4)	55 (6.1)	112 (6.3)
White and black Caribbean	17 (1.9)	15 (1.7)	32 (1.8)
White and black African	3 (0.3)	5 (0.6)	8 (0.5)
White and Asian	8 (0.9)	6 (0.7)	14 (0.8)
Mixed other	7 (0.8)	8 (0.9)	15 (1.8)
Indian	11 (1.2)	10 (1.1)	21 (1.2)
Pakistani	9 (1.0)	6 (0.7)	15 (0.8)
Bangladeshi	2 (0.2)	13 (1.5)	15 (0.8)
Asian other	3 (0.3)	3 (0.3)	6 (0.3)
Black-Caribbean	29 (3.3)	34 (3.8)	63 (3.5)
Black African	8 (0.9)	13 (1.5)	21 (1.2)
Black other	4 (0.5)	3 (0.3)	7 (0.4)
Chinese	3 (0.3)	2 (0.2)	5 (0.3)
Other	12 (1.3)	14 (1.6)	26 (1.5)
More than one option	0	4 (0.4)	4 (0.2)
Missing	9 (1.0)	7 (0.8)	16 (0.9)
Educational qualifications:			
Degree, or equivalent, and higher	201 (22.5)	218 (24.3)	419 (23.4)
A level, vocational level 3 and higher	198 (22.2)	207 (23.0)	405 (22.6)
Below A level or below vocational level 3	230 (25.8)	212 (23.6)	442 (24.7)
Other (eg, foreign qualification)	52 (5.8)	52 (5.8)	104 (5.8)
No formal qualifications	204 (22.8)	199 (22.1)	403 (22.5)
Missing	8 (0.9)	11 (1.2)	19 (1.06)
Occupation:			
Employed	467 (52.3)	468 (52.1)	935 (52.3)
Unemployed	126 (14.1)	116 (12.9)	242 (13.5)
Homemaker	33 (3.7)	44 (4.9)	77 (4.3)
Student	17 (1.9)	22 (2.5)	39 (2.2)
Retired	153 (17.1)	152 (16.9)	305 (17.1)
Long term sick or disabled	26 (2.9)	26 (2.9)	52 (2.9)
Missing	4 (0.5)	8 (0.9)	12 (0.7)
Method of tobacco consumption:			
Manufactured cigarette	615 (68.9)	607 (67.5)	1222 (68.2)
Tobacco roll-up	272 (30.5)	284 (31.6)	556 (31.0)
Cigar	6 (0.7)	8 (0.9)	14 (0.8)
Mean (SD) No of cigarettes/day	18.7 (9.0)	19.1 (9.6)	18.9 (9.3)
Mean (SD) dependence*	5.2 (2.2)	5.2 (2.2)	5.2 (2.2)
Mean (SD) carbon monoxide concentration (ppm)	23.8 (12.8)	23.5 (12.3)	23.7 (12.5)
Mean (SD) longest previous abstinence (days)	358.4 (750.7)	442.3 (993.7)	400.3 (881.4)
Smoking cessation support in past 6 months:			
Yes	304 (34.0)	279 (31.0)	583 (32.5)
No	588 (65.9)	619 (68.9)	1207 (67.4)
Missing	1 (0.1)	1 (0.1)	2 (0.1)

* Fagerstrom test for cigarette dependence, scored 0-10, with higher scores representing greater dependence.

### Drug adherence in preloading arm

Three quarters of participants used the patch daily during the first week and four fifths did so in the subsequent weeks. Overall, 49 (5.5%) people prematurely discontinued preloading; most during the first week of treatment.

We assessed drug used after quit day to support abstinence in those making a quit attempt (table 2). Nicotine patch use was more common in the preloading arm and varenicline use more common in the control arm.

**Table 2:** Drugs used to support smoking cessation among those who made a quit attempt. Values are numbers (percentages)

Treatments	Control arm (n=738)	Preloading arm (n=742)	Total (n=1480)
None	87 (11.8)	61 (8.2)	148 (10.0)
Varenicline	218 (29.5)	164 (22.1)	382 (25.8)
Bupropion	6 (0.8)	12 (1.6)	18 (1.2)
Nicotine patches only	99 (13.4)	169 (22.8)	268 (18.1)
Acute nicotine only	74 (10)	44 (5.9)	118 (8.0)
Combined nicotine	156 (21.1)	170 (22.9)	326 (22.0)
Missing	113 (15.3)	135 (18.2)	248 (16.8)

Percentages add to more than 100% because some people used multiple drugs.

### Primary outcome and secondary outcomes

The primary outcome, biochemically validated abstinence at six months, was achieved by 157/899 (17.5%) of participants in the preloading arm and 129/893 (14.4%) in the control arm, a difference of 3.0% (95% confidence interval −0.4% to 6.4%).

The secondary outcomes showed similar modest differences. At four weeks, 319/899 (35.5%) participants in the preloading arm and 288/893 (32.3%) in the control arm achieved seven day point prevalence. At 12 months, 126/899 (14.0%) participants in the preloading arm and 101/893 (11.3%) in the control arm achieved validated prolonged abstinence. Table 3 presents the primary and secondary outcomes adjusted for centre—that is, the primary analysis.

**Table 3:** Primary analysis of primary and secondary outcomes*

Outcomes	Odds ratio (95% CI)†	P value		Risk ratio (95% CI)‡	P value		Risk difference (95% CI)	P value
Primary outcome:								
6 month Russell standard	1.25 (0.97 to 1.62)	0.08		1.21 (0.98 to 1.50)	0.08		3.02 (−0.37 to 6.41)	0.08
Secondary outcomes:								
4 weeks Russell standard	1.21 (1.00 to 1.48)	0.05		1.14 (1.00 to 1.29)	0.05		4.33 (−0.04 to 8.70)	0.05
4 weeks 7 day point prevalence	1.16 (0.95 to 1.41)	0.15		1.10 (0.97 to 1.25)	0.15		3.22 (−1.15 to 7.59)	0.15
6 months 7 day point prevalence	1.13 (0.90 to 1.41)	0.31		1.10 (0.92 to 1.31)	0.31		1.98 (−1.81 to 5.76)	0.31
12 months Russell standard	1.28 (0.97 to 1.69)	0.08		1.24 (0.97 to 1.58)	0.09		2.71 (−0.37 to 5.78)	0.08
12 months 7 day point prevalence	1.23 (0.97 to 1.54)	0.08		1.17 (0.98 to 1.41)	0.08		3.32 (−0.42 to 7.06)	0.08

* All participants included in analysis and assumed to be smoking if true status was unknown. Denominators were 893 in control arm and 899 in preloading arm.

† Primary form of analysis.

‡ Calculated from odds ratios using adjrr command in Stata.

Adjustment for other predictors of abstinence left the results essentially unchanged, but adjustment for the use of post-quit day varenicline changed the results noticeably; the unadjusted results were statistically significant at four weeks and at six and 12 months. The odds ratio increased from 1.25 (0.97 to 1.62) to 1.34 (1.03 to 1.73), P=0.03 for the primary outcome (table 4 and supplementary table 1).

**Table 4:** Primary and secondary outcomes expressed as risk ratios and risk differences showing the effect of sequential planned adjustment in sensitivity analysis*

Outcomes	Unadjusted			Adjusted†			Adjusted‡			Adjusted§	
	Estimate (95% CI)	P value		Estimate (95% CI)	P value		Estimate (95% CI)	P value		Estimate (95% CI)	P value
**Primary outcome: 6 month Russell standard**											
Estimated risks	17.5 and 14.4										
Risk ratio	1.25 (0.97 to 1.62)	0.08		1.21 (0.98 to 1.50)	0.08		1.21 (0.98 to 1.50)	0.08		1.27 (1.03 to 1.57)	0.03
Risk difference	3.02 (−0.37 to 6.41)	0.08		3.02 (−0.37 to 6.41)	0.08		3.03 (−0.37 to 6.43)	0.08		3.80 (0.41 to 7.18)	0.03
**Secondary outcomes**											
4 weeks Russell standard:											
Estimated risks	36.3 and 31.9										
Risk ratio	1.14 (1.00 to 1.29)	0.05		1.14 (1.00 to 1.29)	0.05		1.14 (1.00 to 1.29)	0.05		1.19 (1.05 to 1.35)	0.007
Risk difference	4.35 (−0.04 to 8.73)	0.05		4.33 (−0.04 to 8.70)	0.05		4.37 (−0.01 to 8.75)	0.05		5.89 (1.60 to 10.19)	0.007
4 weeks 7 day point prevalence:											
Estimated risks	35.5 and 32.3										
Risk ratio	1.10 (0.97 to 1.25)	0.15		1.10 (0.97 to 1.25)	0.15		1.10 (0.97 to 1.25)	0.15		1.15 (1.02 to 1.31)	0.03
Risk difference	3.23 (−1.15 to 7.61)	0.15		3.22 (−1.15 to 7.59)	0.15		3.22 (−1.17 to 7.60)	0.15		4.86 (0.58 to 9.14)	0.03
6 months 7 day point prevalence:											
Estimated risks	22.3 and 20.3										
Risk ratio	1.10 (0.92 to 1.31)	0.31		1.10 (0.92 to 1.31)	0.31		1.10 (0.92 to 1.32)	0.28		1.15 (0.96 to 1.37)	0.13
Risk difference	1.98 (−1.81 to 5.77)	0.31		1.98 (−1.81 to 5.76)	0.31		2.11 (−1.68 to 5.91)	0.28		2.93 (−0.85 to 6.71)	0.13
12 months Russell standard:											
Estimated risks	14.0 and 11.3										
Risk ratio	1.24 (0.97 to 1.58)	0.09		1.24 (0.97 to 1.58)	0.09		1.24 (0.97 to 1.58)	0.09		1.30 (1.02 to 1.66)	0.04
Risk difference	2.71 (−0.37 to 5.78)	0.08		2.71 (−0.37 to 5.78)	0.08		2.66 (−0.43 to 5.75)	0.09		3.31 (0.22 to 6.39)	0.04
12 months 7 day point prevalence:											
Estimated risks	22.4 and 19.0										
Risk ratio	1.17 (0.98 to 1.41)	0.08		1.17 (0.98 to 1.41)	0.08		1.17 (0.98 to 1.41)	0.09		1.21 (1.01 to 1.45)	0.04
Risk difference	3.32 (−0.43 to 7.07)	0.08		3.32 (−0.42 to 7.06)	0.08		3.28 (−0.48 to 7.04)	0.09		3.98 (0.23 to 7.73)	0.04

* All participants included in analysis and assumed to be smoking if true status was unknown. Denominators were 893 in control arm and 899 in preloading arm.

† Adjusted for research centre (primary analysis).

‡ Adjusted for research centre, previous longest abstinence, baseline strength of urges to smoke (both continuous, following analysis plan).

§ Adjusted for research centre, previous longest abstinence, baseline strength of urges to smoke (both continuous, following analysis plan), and varenicline prescribed at +1 week.

### Subgroup analysis

There was no evidence that people who were classified as more dependent on smoking received a greater benefit from preloading. The P values for multiplicative interaction terms for the effect of preloading in those with higher dependence scores and higher exhaled carbon monoxide concentrations were 0.83 and 0.17, respectively. Per unit increase in each variable, the odds ratios were 1.01 (95% confidence interval 0.90 to 1.14) and 1.01 (0.99 to 1.04), respectively.

There was no evidence that people who used varenicline as their post-cessation drug received less benefit from nicotine preloading than people using other cessation drugs. The odds ratios for the effect of preloading compared with control for users of varenicline was 1.42 (0.90 to 2.26) and for non-users was 1.30 (0.95 to 1.77), P=0.74 for the interaction.

### Effect of preloading on urge to smoke

One week into preloading or as control, the urge to smoke had decreased in people using preloading. The mean score on the mood and physical symptom scale-craving subscale at −3 weeks was 2.1 (SD 0.8) for the preloading arm and 2.6 (0.9) for the control arm: baseline adjusted difference −0.5 (95% confidence interval −0.6 to −0.4). One week after quit day, in those who were abstinent, scores on the mood and physical symptom scale-craving subscale were 1.0 (1.0) for the preloading arm and 1.3 (1.0) for the control arm, difference −0.3 (−0.4 to −0.1) and for those who were still trying to achieve abstinence, the corresponding values were 1.3 (1.1), 1.5 (1.1), difference −0.2 (−0.4 to −0.1).

### Adverse events

Spontaneously reported adverse events of moderate or severe intensity were uncommon in both arms. There were eight system or organ class groups where at least 10 participants reported one symptom within that group. Of these, gastrointestinal disorders, general disorders, and nervous system disorders were statistically significantly more common in those using preloading, with absolute differences of 4.0% (2.2% to 5.9%), 2.1% (0.7% to 3.5%), and 4.5% (2.7% to 6.4%), respectively. There were 15 individual symptoms where at least five participants reported that symptom. Of these, nausea occurred in 2.5% (1.1% to 3.8%) more people who were preloading, and vomiting in 1.2% (0.3% to 2.2%). Fatigue was also more common in people preloading, by 0.7% (0.1% to 1.2%), as were well recognised adverse effects of nicotine patches—namely, abnormal dreams 0.9% (0.2% to 1.6%), poor sleep 1.9% (0.9% to 3.0%), and headaches 1.2% (0.3% to 2.2%). Supplementary table 2 presents the full results.

Sixteen serious adverse events occurred during the five week period: eight in the preloading arm and eight in the control arm (odds ratio 0.99, 95% confidence interval 0.36 to 2.75). Of these, one was judged possibly due to preloading: a 64 year old woman in the preloading arm who had an acute coronary syndrome (see supplementary table 3).

One week after baseline, 394 (45.5%) participants in the preloading arm and 271 (32.4%) in the control arm reported at least one symptom of nicotine excess on the symptom questionnaire (P<0.001 for the difference). Of the 12 symptoms, three were statistically significantly more common in the preloading arm: nausea, dizziness, and palpitations. Of these symptoms, the percentages in intervention and control arms with somewhat or very noticeable symptoms were 5.6% and 3.0% for dizziness, 3.9% and 1.9% for palpitations, and 8.1% and 3.1% for nausea (see supplementary figure 1).

## Discussion

In this pragmatic open label trial, there was no strong evidence that four weeks of nicotine patch treatment increased the rate of prolonged abstinence at six months in the primary analysis. Preloading was tested in a clinical setting where smokers could opt to use either nicotine replacement therapy or non-nicotine pharmacotherapies for continued cessation treatment after preloading had ended. In a planned analysis adjusted for varenicline use, there was clearer evidence that preloading increased the likelihood of achieving abstinence. Preloading reduced the intensity of urge to smoke both before and after attempting abstinence, which suggests it is an effective treatment. As 95% of participants continued preloading treatment, 80% using it daily, preloading seems to be well tolerated. Around 1 in 20 people experienced adverse events caused by preloading that were moderate or severe. No evidence was found of an excess of serious adverse events.

### Strengths and limitations of this study

This trial has strengths and limitations. It was considerably larger than previous studies on this topic, thus achieving good precision. The pragmatic design makes the results easier to apply to clinical practice—for example, that staff used their judgment to define tobacco dependence, avoiding the use of arbitrary cut-offs for cigarettes per day, which are not used in clinical practice. Likewise, we included people who had serious coexisting medical conditions, psychiatric disorders, and other substance misuse problems and people from lower socioeconomic groups, reflecting the population of people who seek help to stop smoking. Around 75% of the study population were white British, lower than in England as a whole, reflecting the cities in which we recruited. However, the likely mechanism of action of preloading is that it undermines cigarette dependence and this biological action is likely to apply to any dependent smoker, regardless of her or his ethnic group. Around half of all potential participants who inquired about the trial were not enrolled and this might be thought to indicate poor acceptability of this particular intervention. However, this ratio between inquiries and participation seems to hold in other smoking cessation trials that recruited in the same way but that offered more “benign” interventions, such as St John’s wort, or a behavioural intervention in addition to routine care,^27^ ^28^ so we believe that this is more likely to indicate people’s willingness to quit smoking, quit with support, or attend a schedule of treatment and follow-up visits. While this is unlikely to bias the difference between arms, it may also indicate that not everyone considering supported cessation is prepared to engage with this particular intervention. We used a robust method to assess the occurrence of adverse events. As it is unintuitive for participants who are not receiving treatment to report apparent side effects, we trained staff to inquire regardless. We supplemented this approach with a self completion questionnaire for participants in both arms that concerned symptoms experienced. In the event, both methods revealed similar findings, with nausea emerging as the most common adverse effect caused by preloading, albeit most did not experience it. Our trial was the first trial of preloading to assess adverse events to standards defined by good clinical practice and to record serious adverse events. Only one serious adverse event—acute coronary syndrome—was ascribed by an independent committee blinded to allocation as possibly due to preloading. This was ascribed on the basis that nicotine increases pulse rate, which may predispose to acute coronary syndrome. This participant, however, had stopped using the nicotine patch two days before the event. Nicotine has a half-life of around two hours,^29^ so even with a skin reservoir, nicotine from the patch though not from smoking would have cleared from the blood and thus not be exerting acute pharmacological effects. The UK Committee on the Safety of Medicines reviewed the cardiac safety of nicotine replacement therapy and recommended removal of licence restrictions on its use in people with stable cardiovascular disease and concurrent use while smoking.^30^ This is based on trials in people with cardiovascular disease and large scale observational evidence that shows no evidence of an increased risk.^31^ ^32^ Short term studies show that high dose nicotine patches of up to 63 mg/day while smoking exert no greater effect on the cardiovascular system than does smoking alone.^33^ It therefore seems unlikely that this event was caused by preloading. The open label design is both a strength and a limitation. As a strength, it suggested an effect of preloading that either promoted the use of nicotine patches for use post-cessation or deterred the use of varenicline, or both. In all other trials of preloading, the investigators controlled the choice of post-cessation drug and therefore this effect was not apparent. Arguably, this effect may occur in routine clinical practice and this has important implications for practice. As a limitation, a placebo would have provided more certainty that participants’ expectations were matched evenly by arm. Inequality of expectations might have influenced participants, but is an unlikely cause of the effect on cessation. The Cochrane review of nicotine replacement therapy contrasts trials where participants were randomised to nicotine replacement therapy for post-quit day use or matching placebo; in these studies, the risk ratio was 1.51 (95% confidence interval 1.39 to 1.63) for long term abstinence. In studies without blinding, the risk ratio was similar at 1.58 (1.43 to 1.74).^6^ Perhaps lack of blinding affected participants’ reports of adverse events and urge intensity, although the effects on urge intensity persisted at least a week after the end of treatment. In fact there is evidence that expectations of success were matched in this trial; ratings of confidence in ability to quit one week into preloading/control did not differ significantly by arm. It was also not possible to blind outcome assessors, because the one staff member employed in each centre to perform clinical duties did both recruitment and follow-up. However, smoking abstinence was biochemically validated and this is unlikely to have affected the results.

### Comparison with other studies

Although this trial does not provide strong evidence in itself, other evidence suggests that the optimum management of tobacco dependence includes a period of treatment before a quit attempt. Since inception of this trial, three further trials have published data on short term abstinence, two comparing varenicline preloading and one bupropion with standard use.^8^ ^9^ ^10^ The risk ratios for abstinence in these trials were 2.14 (95% confidence interval 1.14 to 4.00) and 1.35 (0.77 to 2.38) for varenicline (combined 1.78, 1.17 to 2.71) and 1.70 (1.04 to 2.80) for bupropion. Adding our trial to the previous inconclusive meta-analysis of nicotine patch preloading versus standard use gives risk ratio of 1.24 (1.07 to 1.43) for long term abstinence from nicotine patch preloading. Previous trials have reported that preloading reduces the intensity of urges to smoke and smoking consumption in the pre-quit period and that this seems to be part of its mechanism of action.^12^ What we have shown is that, in line with theory, this reduction of intensity of urges before quitting translates into reduced intensity of urge after quit day. Aside from its theoretical importance, this finding could have implications for smoking cessation practice. Some trials have suggested that response to preloading predicts success in quitting,^34^ and, if preloading were to be adopted, it may be sensible to continue preloading only in people who experience reduced urges to quit, although this needs to be confirmed by randomised trials. Our trial is the only one to be carried out in the context where the therapists who prescribed the preloading (our trial team) were different from those who prescribed the post-cessation support (the NHS stop smoking service). Doing so showed that preloading can be effective in this context, but its benefit may have been undermined by reduced varenicline use in those allocated to nicotine preloading. This effect may have occurred because English (National Institute for Health and Care Excellence) guidelines, which state “Do not offer nicotine replacement therapy, varenicline or bupropion in any combination.”^35^ If so, changing this guidance may overcome this problem and nicotine preloading might be effective in such a context.

### Implications for policy and practice

The best estimate of effect is that nicotine preloading could lead to around 3% of people seeking help to quit smoking, achieving prolonged abstinence at 12 months that might not otherwise have done so. This effect may seem small but current 12 months quit rate in the UK specialist cessation services is 8%,^36^ and so an additional 3% would represents a worthwhile improvement. A failed quit attempt is likely to cost someone about 3-5 years of life expectancy.^22^ Thus, around 12 people need to use preloading to gain around a year of life. However, this trial does not provide sufficiently strong evidence to be confident that nicotine preloading is effective, probably because of the reduced use of varenicline that followed preloading. Both observational and randomised trial evidence suggest that varenicline use during the first weeks of abstinence is more effective than nicotine patches alone.^25^ ^36^ ^37^ It therefore seems important to examine how to mitigate this unintended effect.

### Conclusion

Nicotine preloading with a 21 mg/24hr nicotine patch for four weeks seems to be efficacious, safe, and well tolerated, but probably deters the use of varenicline, the most effective smoking cessation drug. If it were possible to overcome this unintended consequence, preloading could lead to a worthwhile increase in long term smoking abstinence.

**What is already known on this topic**

Smoking cessation pharmacotherapy is recommended in the period after a quit day
A 2011 meta-analysis reported that using pharmacotherapy before quit day may increase abstinence, but the data were heterogeneous and evidence of long term benefit was not clear

**What this study adds**

In this trial conducted in a routine health service context, there was no clear evidence that using nicotine patches for four weeks before quit improved long term abstinence
The benefit of preloading, however, may have been masked by reduced use of varenicline in people allocated to preloading, which, being more effective, reduced the overall benefit
After adjusting for this effect, evidence of possible effectiveness was clearer

## Web extra

Extra material supplied by authors

## Notes

Contributions of authors (in alphabetical order):

Writing committee (study design, data analysis, implementation and interpretation, and study write-up): Marcus Munafo, Nicola Lindson, Peter Hajek, Paul Aveyard, Sarah Lewis, and Tim Coleman.

Study management (including recruitment and follow-up): Alia Ataya, Alice Scott, Andy McEwen, Angela Attwood, Anna Phillips, Anne Dickinson, Carmen Wood, Celine Homsey, Clare Randall, Deborah Lycett, Diana Pratt, Doug Coyle, Dunja Przulj, Emma Anderson, Emma Howell, Gurmail Rai, Hayden McRobbie, Jasmine Khouja, Jinshuo Li, Steve Parrott, Jo Perdue, Kate Myers, Katherine Evans, Kathryn Coyle, Kayleigh Easey, Khaled Ahmed, Lindsey Lacey, Lizzy Dann, Marcus Munafo, Mark Allen, Megan Fluharty, Megan Hurse, Mike Healy, Miriam Banting, Natalie Bisal, Nicola Lindson, Paul Aveyard, Peter Hajek, Rachel Adams, Rebecca Anderson, Rhona Alekna, Sarah Lewis, Sarah Tearne, Shahnaz Khan, Sophie Duncombe, Sophie Orton, Subhash Pokhrel, Therese Freuler, and Tim Coleman.

Chief investigator: Paul Aveyard. Trial statistician: Sarah Lewis. Guarantors: Paul Aveyard and Sarah Lewis.

Independent trial steering committee: Michael Ussher (chair), Dan Griffin, Helen Stokes-Lampard, Jane Wright, Lion Shahab, Rumana Omar, Tess Harris. There was no data monitoring and ethics committee.

Ethical approval: This trial was approved by National Research Ethics Service Committee East Midlands-Leicester (No 12/EM/0014).

Data sharing: The authors will share data that is in the interests of public health and provided they have not planned similar analyses.

Transparency: The corresponding author (Paul Aveyard) affirms that this manuscript is an honest, accurate, and transparent account of the study being reported; that no important aspects of the study have been omitted; and that any discrepancies from the study as planned have been explained.
