# Supplementary material for: Effects on abstinence of nicotine patch treatment before quitting smoking: parallel, two arm, pragmatic randomised trial
Source: BMJ. 2018 Jun 13;361:k2164. doi: 10.1136/bmj.k2164 (PMC5998048; doi:10.1136/bmj.k2164)
Supplement: Supplementary file 1 — Supplementary information: additional tables and figure [file avep041696.ww1.pdf]

Online Appendix

Table 1 Primary and secondary smoking cessation outcomes presented as odds ratios for preloading arm versus control (primary analysis)*

Outcome	Unadjusted		Adjusted Ɨ		Adjusted β		Adjusted α	
	OR (95%CI)	p	OR (95%CI)	p	OR (95%CI)	p	OR (95%CI)	p
**Primary outcome:**								
6 month Russell standard	1.25 (0.97 to 1.62)	0.08	1.25 (0.97 to 1.62)	0.08	1.26 (0.97 to 1.62)	0.08	1.34 (1.03 to 1.73)	0.03
**Secondary outcomes**								
4 weeks Russell standard	1.21 (1.00 to 1.48)	0.05	1.21 (1.00 to 1.48)	0.05	1.22 (1.00 to 1.48)	0.05	1.32 (1.08 to 1.62)	0.007
4 week 7-day point prevalence	1.16 (0.95 to 1.41)	0.15	1.16 (0.95 to 1.41)	0.15	1.16 (0.95 to 1.41)	0.15	1.26 (1.03 to 1.54)	0.03
6 months 7-day point prevalence	1.13 (0.90 to 1.41)	0.31	1.13 (0.90 to 1.41)	0.31	1.14 (0.90 to 1.43)	0.28	1.20 (0.95 to 1.51)	0.13
12 months Russell standard	1.28 (0.97 to 1.69)	0.08	1.28 (0.97 to 1.69)	0.08	1.27 (0.96 to 1.69)	0.09	1.36 (1.02 to 1.80)	0.04
12 months 7-day point prevalence	1.22 (0.97 to 1.54)	0.08	1.23 (0.97 to 1.54)	0.08	1.22 (0.97 to 1.54)	0.09	1.28 (1.01 to 1.62)	0.04

*all participants included in the analysis and assumed to be smoking if true status was unknown and the denominators were 893 control arm, 899 intervention arm

Ɨ adjusted for research centre- the primary analysis

β adjusted for research centre, previous longest abstinence (days, continuous), baseline strength of urges to smoke (continuous, as per analysis plan)

α adjusted for research centre, previous longest abstinence (days, continuous), baseline strength of urges to smoke (continuous, as per analysis plan), varenicline prescribed +1 week

Table 2 Reported adverse events of moderate or severe intensity in the intervention and control arms

Event	Control (N = 860)	Intervention (N = 880)	Percentage-point difference (95%CI)
Gastrointestinal disorders	19 (2.2%)	55 (6.2%)	4.0 (2.2 to 5.9)
Abdominal pain	3 (0.3%)	6 (0.7%)	0.3 (-0.3 to 1.0)
Diarrhoea	3 (0.3%)	8 (0.9%)	0.6 (-0.2 to 1.3)
Nausea	8 (0.9%)	30 (3.4%)	2.5 (1.1 to 3.8)
Vomiting	3 (0.3%)	14 (1.6%)	1.2 (0.3 to 2.2)
General disorders	11 (1.2%)	30 (3.3%)	2.1 (0.7 to 3.5)
Asthenia	5 (0.6%)	10 (1.1%)	0.6 (-0.3 to 1.4)
Fatigue	0 (0.0%)	6 (0.7%)	0.7 (0.1 to 1.2)
Injuries to poisoning to and procedural complications	8 (0.9%)	4 (0.5%)	-0.5 (-1.2 to 0.3)
Musculoskeletal and connective disorders	7 (0.8%)	10 (1.1%)	0.3 (-0.6 to 1.2)
Nervous system	16 (1.9%)	56 (6.4%)	4.5 (2.7 to 6.4)
Abnormal dreams	1 (0.1%)	9 (1.0%)	0.9 (0.2 to 1.6)
Dizziness	6 (0.7%)	15 (1.7%)	1.0 (0.0 to 2.0)
Headache	3 (0.3%)	14 (1.6%)	1.2 (0.3 to 2.2)
Poor quality sleep	3 (0.3%)	20 (2.3%)	1.9 (0.9 to 3.0)
Psychiatric	7 (0.8%)	17 (1.9%)	1.1 (0.0 to 2.2)
Depressed mood	4 (0.5%)	5 (0.6%)	0.1 (-0.6 to 0.8)
Respiratory	21 (2.4%)	15 (1.7%)	-0.7 (-2.1 to 0.6)
Chest infection	4 (0.5%)	1 (0.1%)	-0.3 (-0.8 to 0.2)
Influenza like illness	3 (0.3%)	7 (0.8%)	0.4 (-0.3 to 1.2)
Nasopharyngitis	7 (0.8%)	4 (0.5%)	-0.4 (-1.1 to 0.4)
Skin and subcutaneous tissue disorders	4 (0.5%)	7 (0.8%)	0.3 (-0.4 to 1.1)
Skin irritation	2 (0.2%)	5 (0.6%)	0.3 (-0.3 to 0.9)

Table reports proportion of people reporting adverse events for any system or organ class term with at least 10 or more participants reporting any single event and any preferred term that had at least five participants reporting it. The denominator includes all who participated either at -3 or +1 week.

Table 3 Serious adverse events

Age and gender	Trial arm	Relevant medical history	Event
68-year-old man	Intervention	Chronic myeloid leukaemia	Hospitalised with chest infection
85-year-old woman	Intervention	Osteoporosis	Hospitalised with pelvic fracture following accidental fall
72-year-old woman	Intervention	None	Hospitalised for aspiration of malignant pleural effusion
65-year old woman	Intervention	Cardiovascular disease, hypertension, pacemaker and history of blackouts	Hospitalised for a blackout
27-year-old woman	Intervention	Psychotic illness, illicit drug use	Hospitalised for psychotic episode following period of illicit drug use leading to anxiety attacks
64-year-old woman	Intervention	None	Acute coronary syndrome
54-year-old woman	Intervention	Angina	Hospitalised with acute coronary syndrome or non-cardiac chest pain
45-year-old woman	Intervention	Self-harming	Hospitalised for increased self-harming and suicidal ideation
68-year-old man	Control	Reflux oesophagitis	Hospitalised with cancer of the oesophagus
55-year-old woman	Control	COPD and type 2 diabetes	Death due to COPD
59-year-old man	Control	Alcohol dependence	Death due to accidental house fire
52-year-old man	Control	Two previous hernia repairs	Hospitalised for hernia repair
47-year-old woman	Control	None	Hospitalised for pyelonephritis
38-year-old woman	Control	Asthma	Hospitalised with chest infection
64-year-old woman	Control	COPD	Exacerbation of COPD
25-year-old man	Control	None	Pneumonia

Figure 1 Prevalence and severity of symptoms reported in the last 24 hours reported one week into preloading or control

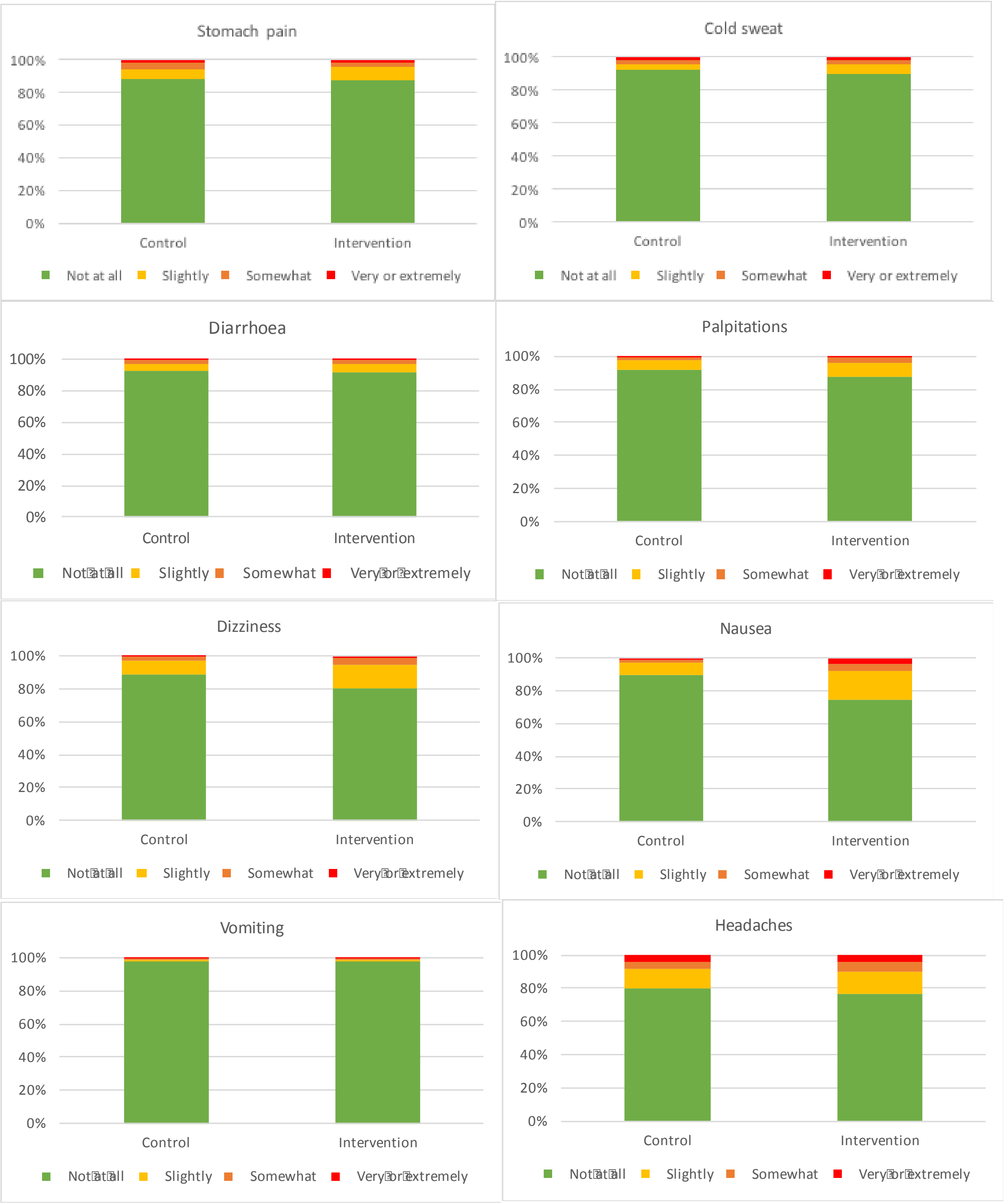

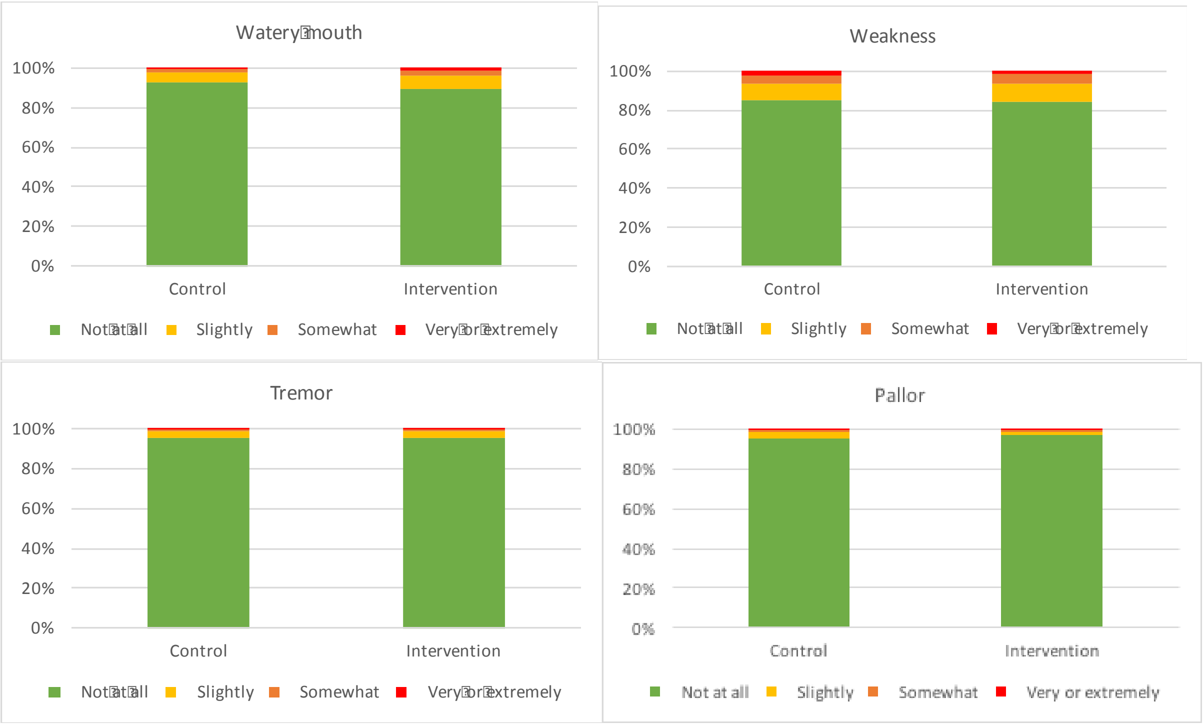
Watery mouth
100%
80%
60%
40%
20%
0%
Control
Intervention
Not at all
Slightly
Somewhat
Very or extremely
Weakness
100%
80%
60%
40%
20%
0%
Control
Intervention
Not at all
Slightly
Somewhat
Very or extremely
Tremor
100%
80%
60%
40%
20%
0%
Control
Intervention
Not at all
Slightly
Somewhat
Very or extremely
Pallor
100%
80%
60%
40%
20%
0%
Control
Intervention
Not at all
Slightly
Somewhat
Very or extremely
